# Advances in 4-Hydroxyphenylacetate-3-hydroxylase Monooxygenase

**DOI:** 10.3390/molecules28186699

**Published:** 2023-09-19

**Authors:** Kai Yang, Qianchao Zhang, Weirui Zhao, Sheng Hu, Changjiang Lv, Jun Huang, Jiaqi Mei, Lehe Mei

**Affiliations:** 1 Department of Chemical and Biological Engineering, Zhejiang University, Hangzhou 310027, China; kayyoung@zju.edu.cn; 2 School of Biological and Chemical Engineering, Ningbo Tech University, Ningbo 315100, China; zqccome@163.com (Q.Z.); genegun@zju.edu.cn (S.H.); 3 Department of Chemical and Biological Engineering, Zhejiang University of Science and Technology, Hangzhou 310023, China; yangtzelv@zust.edu.cn (C.L.); huangjun@zust.edu.cn (J.H.); 4 Hangzhou Huadong Medicine Group Co., Ltd., Hangzhou 310011, China; meijiaqi@eastchinapharm.com; 5 Jinhua Advanced Research Institute, Jinhua 321019, China

**Keywords:** biocatalysis, 4-hydroxyphenylacetate-3-hydroxylase monooxygenase, catechols, structural property, protein engineering

## Abstract

Catechols have important applications in the pharmaceutical, food, cosmetic, and functional material industries. 4-hydroxyphenylacetate-3-hydroxylase (4HPA3H), a two-component enzyme system comprising HpaB (monooxygenase) and HpaC (FAD oxidoreductase), demonstrates significant potential for catechol production because it can be easily expressed, is highly active, and exhibits *ortho*-hydroxylation activity toward a broad spectrum of phenol substrates. HpaB determines the *ortho*-hydroxylation efficiency and substrate spectrum of the enzyme; therefore, studying its structure–activity relationship, improving its properties, and developing a robust HpaB-conducting system are of significance and value; indeed, considerable efforts have been made in these areas in recent decades. Here, we review the classification, molecular structure, catalytic mechanism, primary efforts in protein engineering, and industrial applications of HpaB in catechol synthesis. Current trends in the further investigation of HpaB are also discussed.

## 1. Introduction

**Figure 1:**
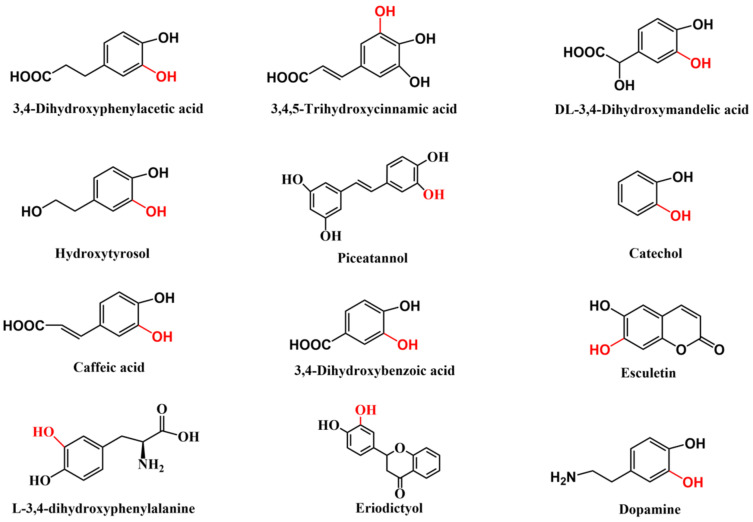
Typical examples of natural products and drugs with catechol substructure (The red represents the position of *ortho*-hydroxylation.).

The catechol structure entity is present in numerous important natural products and bioactive molecules [1] (Figure 1), which typically have excellent physiological functions, such as anti-oxidation [2], anti-inflammatory [3], antiviral [4], and anticancer properties [5], as well as the ability to improve blood circulation [6]. Over 300,000 compounds containing catechol motifs bear useful pharmacological activities [7]. Therefore, these catechol-containing molecules have important applications in the food, pharmaceutical, and cosmetics industries [8]. Moreover, catecholic compounds are widely used to prepare various functional materials, owing to their excellent adhesion, chemical reactivity, metal ion chelation capacity, oxidation–reduction activity, biocompatibility, and degradability [9,10]. Therefore, producing catechol-containing compounds using a simple, efficient, and economical approach is essential. The *ortho*-hydroxylation of phenols is an essential step in catechol synthesis [11]. Although several chemical *ortho*-hydroxylation methods, such as the classical methodology that involves *ortho*-formylation of phenols followed by Dakin oxidation, have been developed [12,13], they generally have difficulties, such as poor selectivity, multistep procedures, harsh reaction conditions, and environmental pollution [14]. To overcome these issues, biological *ortho*-hydroxylation methods with high regioselectivity, mild reaction conditions, and environmental friendliness have been widely investigated [15,16,17].

Currently, three classes of enzymes can perform *ortho*-hydroxylation of single phenolic substances: cytochrome P450 hydroxylase (EC 1.14.14.1) [18,19], phenol oxidase, particularly tyrosinase (EC 1.14.18.1) [20,21], and 4-hydroxyphenylacetate-3-hydroxylase (4HPA3H; EC 1.14.14.9) [22]. In past decades, P450 hydroxylases have been the primary group of enzymes explored and engineered for the *ortho*-hydroxylation of aromatic compounds. However, low catalytic activity is the most commonly encountered problem for P450 enzymes conducting *ortho*-hydroxylation due to its intrinsic catalytic mechanism [16,23]. Phenol oxidase results in catechols via the conversion of phenols to quinones. This requires the subsequent reduction in quinones back to catechols by a reducing agent, such as ascorbic acid. However, this approach has several disadvantages, including pronounced instability of the enzyme, particularly in the presence of molecular oxygen, and the enzyme cannot be used as a synthetic component in metabolic engineering to realize *ortho*-hydroxylation because of the need for a reducing agent. In addition, certain phenol oxidases are reported to be inactivated by phenols and ascorbic acid [24,25,26]. In contrast, 4HPA3H is the preferred and promising enzyme for the development of catalysts for the preparation of catechols because it can be easily expressed, has high activity, and exhibits *ortho*-hydroxylation specificity toward a broad spectrum of substrates [27,28,29]. For example, the 4HPA3H of *Acinetobacter baumannii* can catalyze a series of 4-hydroxyphenylacetate (4HPA) analogs, such as 3,4-dihydroxybenzoic acid, 4-hydroxybenzoic acid, 4-nitrophenol, and others [30]. The 4HPA3H from *Escherichia coli* shows a broader substrate spectrum. It can not only catalyze 4HPA analogs but also a series of large or complex phenolic compounds, such as naringenin, afzelechin, kaempferol, dihydrokaempferol, resveratrol, 4-halophenols [15,31,32], and phenolamines, such as tyramine [33].

**Figure 2:**
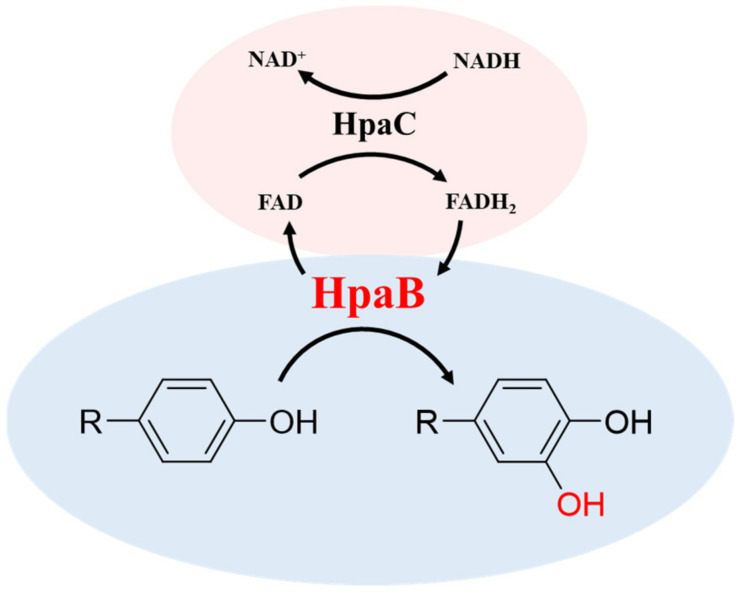
Scheme of the 4HPA3H-intiated reaction.

4HPA3H is a two-component system comprising HpaB (monooxygenase) and HpaC (FAD oxidoreductase) that was initially identified as the first enzymatic step for 4HPA degradation in *E. coli* [22,34]. HpaB is the large component, ranging in size from 39 to 63 KDa, and has been characterized as an FADH_2_-utilizing monooxygenase. HpaC is the small component, with a molecular weight of 16–22 KDa, and is an NAD(P)H-flavin oxidoreductase that couples factors and supplies FADH_2_ to HpaB by consuming NAD(P)H. Therefore, the 4HPA3H-initiated reaction (Figure 2) requires the supply of NAD(P)H to regenerate coenzyme FADH_2_ [22,30,35].

HpaB determines the *ortho*-hydroxylation efficiency and substrate spectrum of the 4HPA3H catalytic system. Therefore, studying its structure–activity relationship and improving its catalytic properties are important; increasing efforts having been made [36,37]. Herein, we review the classification, molecular structure, catalytic mechanism, protein engineering, and industrial applications of HpaB in catechol synthesis. These results enrich the biocatalytic repertoire of HpaB and broaden its future applications and developments.

## 2. Classification of HpaB

**Figure 3:**
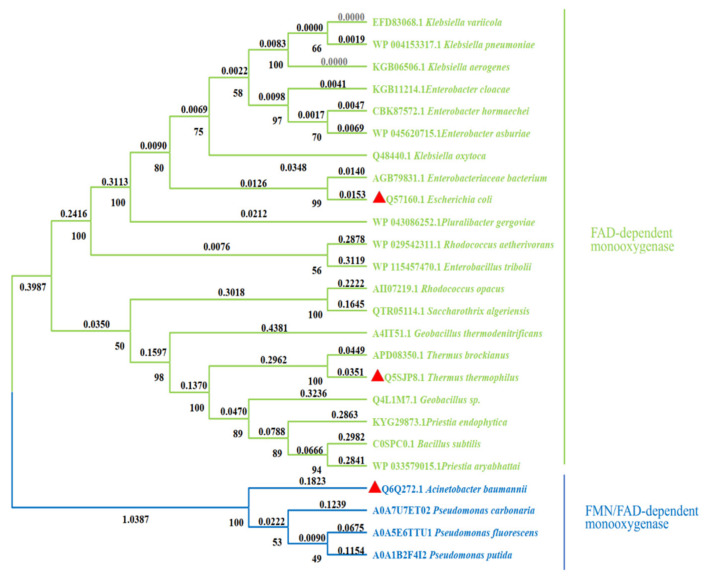
Phylogenetic tree of different HpaB sources. The red triangle represents the HpaB whose crystal structure has been resolved. (Phylogenetic tree was constructed by using MEGAX based on the neighbor-joining algorithm [48]).

HpaB is found in various microorganisms, such as *A. baumannii* [38], *E. coli* [39], *Thermus thermophilus* [40], *Pseudomonas aeruginosa* [41], *Rhodococcus opacus* [42], *Thermophile geobacillus* [43], and *Klebsiella pneumonia* [44]. HpaB from different species can be divided into FAD- and FAD/FMN-dependent monooxygenases [45] (Figure 3). Several primary differences are noted between these two types of enzymes. First, FAD is buried in the FAD-dependent monooxygenases. In contrast, only the isotetrameric ring part of FAD/FMN is buried in the FAD/FMN-dependent monooxygenases, while the rest is solvent-accessible, which is the reason that both the FAD and FMN are available for use [46]. Second, the monomers in FAD-dependent monooxygenases, such as *Tt*HpaB and EcHpaB, contain four domains, and the domain at the C terminal is similar to a tail. In contrast, the monomers in FAD/FMN-dependent monooxygenases, such as AbHpaB, contain only three domains and lack a C-terminal tail structure like FAD-dependent monooxygenases [11]. Third, in FAD-dependent monooxygenases, a conformational change occurs in the loop region of the core domain when FAD binds [45,47], whereas this change does not occur in FAD/FMN-dependent monooxygenases [46]. Finally, FAD-dependent monooxygenases have highly conserved catalytic key sites: Arg100-Tyr104-His142 (*Tt*HpaB numbering). In contrast, FAD/FMN-dependent monooxygenase does not have these corresponding sites; however, His396 could be the catalytic key site (*Ab*HpaB numbering) [45,46].

## 3. HpaB Structure and Catalytic Mechanism

**Figure 4:**
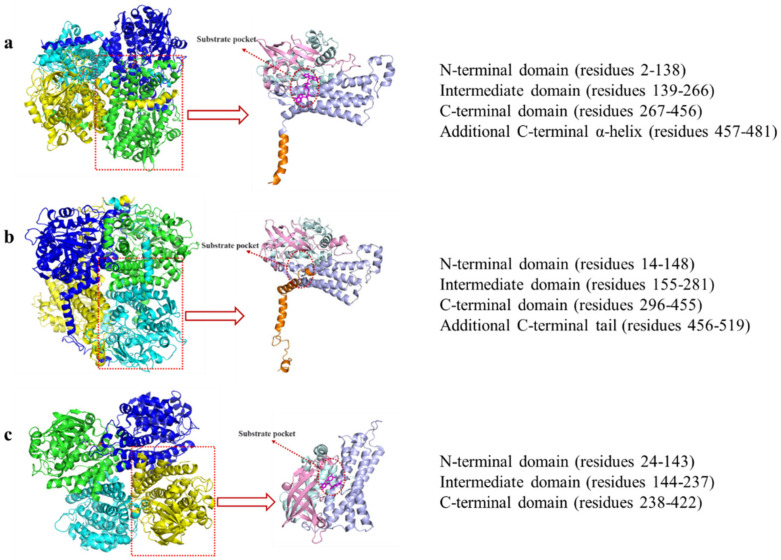
Tetramer and monomer structures of (**a**) *Tt*HpaB (PDB: 2YYJ), (**b**) *Ec*HpaB (PDB: 6QYI), and (**c**) *Ab*HpaB (PDB: 2JBT). The N-terminal domain is pale cyan, intermediate domain is pink, C-terminal domain is light blue, additional C-terminal α-helix is orange, and FAD and FMN is magenta.

Nowadays, three HpaB crystal structures from different species have been identified: *T. thermophilus* HB8 (*Tt*HpaB, PDB: 2YYJ), *E. coli* (*Ec*HpaB, PDB: 6QYI), and *A. baumannii* (*Ab*HpaB, PDB: 2JBT) (Figure 4). The three are homotetramers. Among homotetramers, HpaB is active as a homodimer and can bind and stabilize reduced FAD in the absence of a substrate to prevent autooxidation of the co-substrate [11,45,46,47]. The overall folding of three HpaBs is similar. However, considerable deviations are observed in the subunit structures between *Ab*HpaB (FAD/FMN-dependent type) and the other two (FAD-dependent type) [11].

**Figure 5:**
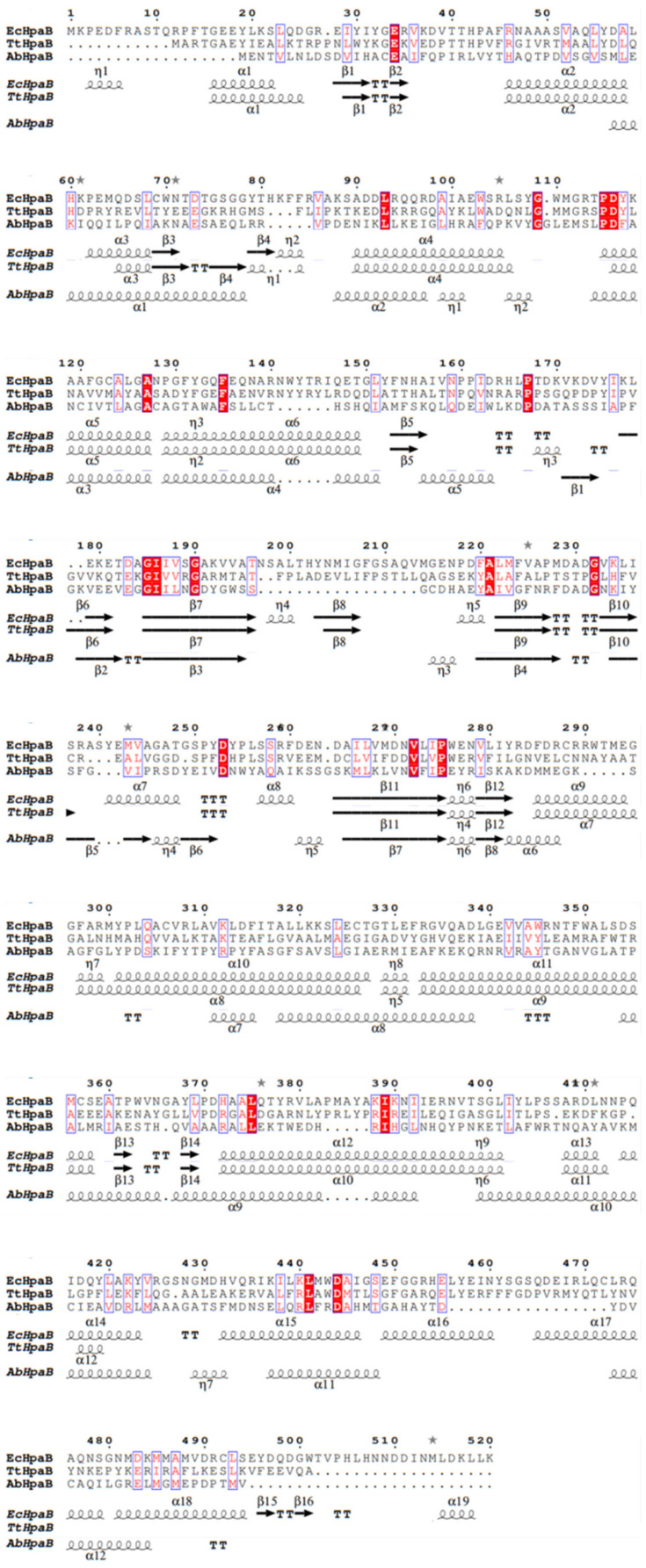
Structure-based sequence alignment of HpaBs from different bacteria. The secondary structural elements are indicated at the bottom of the alignment (Alignment was performed by using ESPript 3 [50]).

Kim et al. determined the crystal structure of *Tt*HpaB [45] (Figure 4a), which was the first structure-solved FAD-dependent HpaB. It is a tetramer comprising four monomers arranged in a dimeric form. The monomer has 481 amino acids and its structure can be divided into four domains: the N-terminal (residues 2–138), intermediate (residues 139–266), and C-terminal domain (residues 267–456), as well as an additional C-terminal α-helix tail (residues 457–481) which extends its α-helix onto the surface of another dimer and could aid in stabilization [45]. A cave at the interspace of the three domains forms the substrate pocket that binds with FAD and the substrate [45,47] (Figure 4a), which consists of β5-β6 and β8-β9 loops (Figure 5). When binding with FAD, the β8-β9 loop in the intermediate domain undergoes a conformational change [45]. The cavity is large enough for the solvent and provides space for the substrate and oxygen following binding of the co-substrate [47]. Another loop in the intermediate domain protects the active site from the solvent after binding to the substrate and the substrate is placed on the *re* face of the FAD isoalloxazine ring [47]. The catalytic key site of *Tt*HpaB in 4HPA comprises Arg100, Tyr104, and His142 (Arg100 and Tyr104 are located in the helix α5 and His142 is located in the β5-β6 loop). These amino acids form hydrogen bonding interactions with the hydroxyl group of 4HPA, play a key role in catalysis and proton transfer, and are highly conserved in the homologous sequence. In addition, S197 and T198, located in the β8-β9 loop, are hydrogen-bonded to the carboxyl group of 4HPA, which play an important role in immobilizing the tail of 4HPA [45].

The crystal structure of *Ec*HpaB, which is FAD-dependent and similar to that of *Tt*HpaB, was resolved by Shen et al. [29] and Deng et al. [11] (Figure 4b). Although *Ec*HpaB exhibits a low sequence identity (26.96%) with *Tt*HpaB, the crystal structures of the two enzymes have a high degree of similarity, with a root mean square deviation of 1.05 Å [11]. *Ec*HpaB is also a tetramer with two homodimers, whose subunit comprises 520 amino acid residues, and a similar subunit domain composition to that of *Tt*HpaB. Deng et al. superimposed the structures of *Tt*HpaB-FAD-4HPA and *Ec*HpaB-FAD and found that the active center of *Ec*HpaB is located near the β8-β9 loop (207–217, *Ec*HpaB numbering) [11]. A major feature of the *Ec*HpaB dimer is the C-terminal tails which are formed by residues 456–519 (α16, α17, α18, α19, β15, and β16) [29] (Figure 5). The C-terminal helical arms interact with their dimer partner. Nine intermolecular hydrogen bonds between the arm and its counterpart help stabilize the dimer [29]. In addition to the results of sequence alignment and molecular docking, the phenol group in the head of 4HPA is anchored in the binding site by hydrogen bonding of its phenol hydroxyl group to residues Arg113, Tyr117, and His155 (*Ec*HpaB numbering), which are structurally conserved in *Tt*HpaB (Arg100, Tyr104, and His142, *Tt*HpaB numbering) [29].

The size and configuration of the binding pocket in FAD-dependent HpaB has a significant influence on the catalytic activity of the enzyme toward different substrates. In a recent study, Wang et al. [42] showed that *Ro*HpaB (from *R. opacus*, the sequence similarity between *Ro*HpaB and *Ec*HpaB is as high as 51.7%) has a larger substrate binding pocket than that of *Ec*HpaB and *Tt*HpaB. The chain length of the 212–222 loop (*R. opacus* numbering) was shorter than *Ec*HpaB (207–217 loop, *Ec*HpaB numbering) and *Tt*HpaB (193–213 loop, *Tt*HpaB numbering), which enables *Ro*HpaB to catalyze the large substrate (naringenin) in a more efficient way than the other two HpaBs. In addition, *Ec*HpaB exhibits higher activity, especially toward large substrates compared to *Tt*HpaB, because of the notable differences observed in the 11-residue 207–217 loop (*Ec*HpaB numbering) connecting strands β8 and β9 between *Ec*HpaB and *Tt*HpaB.

Alfieri et al. determined the crystal structure of *Ab*HpaB [46] (Figure 4c), which is also a tetramer comprising four identical subunits. Each subunit contains 422 amino acids that fold into three domains: N-terminal structural domains (residues 24–143), β-fold (residues 144–237), and C-terminal structural domains (residues 238–422) [46,49]. Notably, the monomer of *Ab*HpaB does not have a tail at the C-terminal domain like that of the FAD-dependent HpaB (*Tt*HpaB or *Ec*HpaB) (Figure 4c). A cave in the interspace of the three domains enables the binding of reduced FMN/FAD to the substrate. The active sites of the catalytic pocket are located at the junction of β-barrels or α-helices and surrounded by various loops, β-barrels, and α-helices, including Phe266 (α8), Arg263 (α7), His120 (α4-α5 loop), Ser146 (β1-β2 loop), and His396 (α11-α12 loop) (Figure 5). Unlike the FAD-dependent monooxygenases mentioned above, when FMN binds to *Ab*HpaB, its conformation does not change. When 4HPA binds to *Ab*HpaB, the angle of Phe266 undergoes an approximate 280° turn, forming a substrate-binding pocket [46]. In addition, Arg263 can form a hydrogen bond with the carboxyl group of 4HPA, which has a stabilizing effect on its tail. His120 and Ser146 have hydrogen bond interactions with the phenolic hydroxyl group of 4HPA, which immobilizes its head. His396 may play two roles in substrate catalysis. First, it can act as a proton donor for the reaction of reduced flavins with oxygen. Second, it can be involved in the protonation of C4a-hydroxyflavins [46].

**Figure 6:**
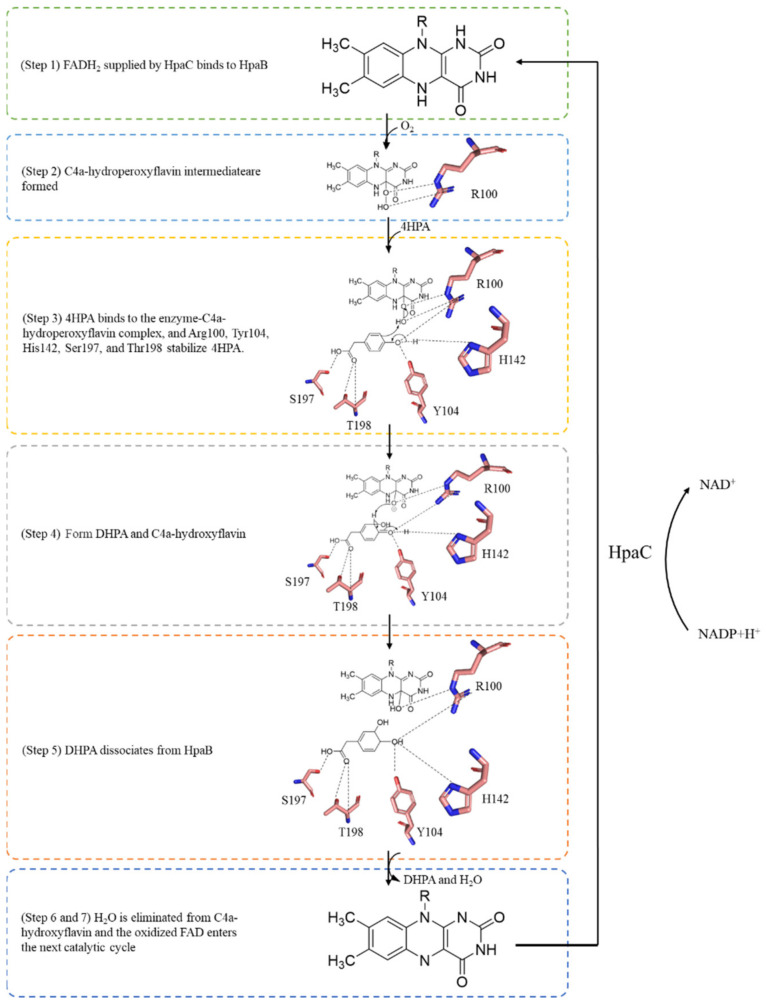
Proposed mechanism of the *Tt*HpaB catalytic reaction.

Based on an analysis of the crystal structure of *Tt*HpaB, the catalytic mechanism of HpaB explained by Kim et al. is as follows [45] (Figure 6): (Step 1) a reduced FAD (FADH_2_) supplied by HpaC binds to HpaB, which forms the substrate-binding site. (Step 2) FADH_2_ accepts an oxygen molecule at the C4a position of the isoalloxazine ring to form a C4a-hydroperoxyflavin intermediate stabilized by Arg100. (Step 3) 4HPA binds to the enzyme-C4a-hydroperoxyflavin complex. Arg100, Tyr104, His142, Ser197, and Thr198 stabilize 4HPA. His142 activates 4HPA via the deprotonation of its 4-hydroxyl group. The hydroxyl group of the C4a-hydroperoxyflavin intermediate is introduced at the *ortho* position of 4HPA by electrophilic attack, yielding the dienone form of the product. (Steps 4 and 5) Re-aromatization of the dienone yields 3,4-dihydroxyphenylacetate (DHPA) and C4a-hydroxyflavin. (Step 6) DHPA dissociates from HpaB. (Step 7) An H_2_O molecule is eliminated from C4a-hydroxyflavin. The oxidized FAD is released from HpaB and recycled into HpaC for reduction for the next catalytic cycle [45,51]. In FAD/FMN-dependent HpaB monooxygenases, such as *Ab*HpaB, FMNH_2_ can be used to form a C4a-hydroperoxyflavin intermediate [51]. When 4HPA binds to the enzyme, the ethoxy of Phe266 moves to enable 4HPA to enter the pocket and then Arg263, His120, and Ser146 in *Ab*HpaB fix 4HPA. Moreover, His396 might be involved in the protonation of C4a-hydroxyflavin. According to the mechanism, this catalytic reaction requires NAD(P)H to regenerate the coenzyme FADH_2_ [22,30,35].

## 4. Molecular Modification of HpaB

HpaB has demonstrated the significant potential for application in the synthesis of catechol-containing compounds. However, its catalytic activity toward certain unnatural substrates, particularly large phenolic compounds, is unsatisfactory for practical applications. For example, the catalytic capacity of wild type *Ec*HpaB decreased with the increased substrate molecular size and structural complexity [29]. Therefore, improving the performance of enzymes and expanding their substrate spectrum through protein engineering has been extensively investigated in recent decades (Table S1). Among the practices, the β8-β9 loop of HpaB (residues 207–217, *Ec*HpaB numbering) within the entrance to the substrate binding pocket is the most popular region for this purpose. By constructing seven mutants with different amino acid sequences and hypothesized secondary structures in this loop and determining their catalytic activities toward different substrates, Shen et al. found both smaller amino acid residues and the flexibility of the loop structure are of key importance to the activity of *Ec*HpaB toward larger substrates. They developed an XS6 mutant (F208S/A211D/Q212L/V213G/M214S/E216S/N217D) with smaller amino acid residues and random coil structures within the loop exhibited increased activity toward larger substrates. For example, the activity of this mutant toward naringenin enhanced about 56% compared with the wild type. [29]. In addition, by establishing and analyzing a molecular model of *Ec*HpaB with 4HPA, Chen et al. found that S210 and A211 in the 207–217 loop (*Ec*HpaB numbering) are likely to stabilize the tail of 4HPA and determine substrate selectivity. Accordingly, they chose S210, A211, and the neighboring site Q212 for simultaneous saturation mutagenesis. Three mutants, H7 (S210T/A211M/Q212G), A10 (S210T/A211L/Q212E), and D11 (A211G/Q212Y), were identified using high-throughput screening. H7 exhibited a 17-fold increase in tyrosol activity and a remarkable 271-fold increase in tyramine activity. A10 showed a 19-fold increase in tyrosol activity compared to that of the wild-type but low activity toward tyramine. In contrast, D11 exhibited 386-fold higher activity on tyramine than the wild-type but low activity toward tyrosol. The authors hypothesized that the significantly higher catalytic activity of mutant H7 for both tyrosol and tyramine than that of wild-type could be attributed to the reduced distance between the substrate and FAD [33]. Similarly, Yao et al. found that the same key sites S210, A211, and Q212 affect the catalytic activity of *Ec*HpaB toward tyrosine. They also performed saturation mutagenesis at these sites and discovered a mutant (S210F/A211K/Q212F), which showed a 12-fold increase in catalytic activity toward tyrosine compared with that of the wild-type [39]. In our laboratory, we identified sites A211 (located in 207–217 loop) and I157 (located in another loop of the substrate pocket) as the key sites affecting the catalytic activity of *Ec*HpaB toward resveratrol by molecular docking and alanine scanning of the substrate pocket. By the saturation mutation of the two sites, we found that the catalytic activity toward resveratrol increased 4.7-fold in the double mutant I157L/A211D compared to that in the wild type. Molecular dynamics simulations showed the flexibility of the G209-E216 and P161-V171 loops in the substrate pockets of the mutant decreased compared with that of the wild type [52]. By performing alanine scanning on the 212–222 loop (*Ro*HpaB numbering, which is corresponding to the loop 207–217 of *Ec*HpaB), Wang et al. found that the *K*_cat_/*K*_m_ of naringenin catalyzed by mutant^Y215A^ increased by 25.3 times relative to the wild-type, which was mainly because this mutation brought the substrate closer to the catalytic triad of *Ro*HpaB (Arg119-Tyr123-His161, *Ro*HpaB numbering) and the smaller residue at the 215th site could make a bigger space in the catalytic domain for naringenin binding [42]. Therefore, both smaller amino acid residues and flexibility of the 207–217 loop (*Ec*HpaB numbering) are beneficial for the HpaB to accept larger substrates.

In addition to the 207–217 loop (*Ec*HpaB numbering), other loops in the substrate pocket also have an influence on the catalytic activity of HpaB toward different substrates. By directed evolution of the *Ec*HpaB mutant^S210T/A211L/Q212E^ (developed in Chen’s study [33]) using error-prone PCR, Qi et al. developed a highly efficient variant HpaB^TLEH^ (S210T/A211L/Q212E/Y282H) to catalyze tyrosol. The recombinant *E. coli* YMG5*R-HpaB^TLEH^C strain (co-expressing HpaB^TLEH^ and *Ec*HpaC) could produce 48.2 mM hydroxytyrosol from 50 mM tyrosol, which exhibited a transformation rate 2.2 and 2.0 times higher than that of the strain YMG5*R-HpaB^TLE^C (expressing HpaB mutant^S210T/A211L/Q212E^ and *Ec*HpaC) and strain YMG5*RHpaBC (expressing wild-type HpaB and *Ec*HpaC), respectively. The increased catalytic efficiency of HpaB^TLEH^ toward tyrosol might be because the 282H causes stronger salt bridges between Arg283 and Asp219, which helped the substrate to be better oriented at the active site and had a higher chance of catalysis to hydroxytyrosol [37].

Wild-type *Ab*HpaB could catalyze the *ortho*-hydroxylation of 4HPA and *p*-coumaric acid to 2-(3,4-dihydroxyphenyl) acetic acid (3,4-DHPA) and caffeic acid, respectively, similar to that of *Ec*HpaB and *Tt*HpaB. However, it could also initiate the *ortho*-hydroxylation of 3,4-DHPA and caffeic acid to 2-(3,4,5-trihydroxyphenyl) acetic acid (3,4,5-THPA) and 3,4,5-trihydroxycinnamic acid (3,4,5-THCA), respectively. Although wild-type *Ab*HpaB showed considerable ability to convert 4HPA into 3,4,5-THPA, it was unable to synthesize 3,4,5-THCA from *p*-coumaric acid efficiently. In order to overcome this, Dhammaraj et al. developed a mutant Y398S based on the rational design and site-directed mutagenesis of *Ab*HpaB, which could catalyze the complete bioconversion of *p*-coumaric acid to 3,4,5-THCA within 180 min and was significantly improved from the wild-type reaction. The significant reduction in the side chain at the 389th site created more space, which better accommodated the binding of *p*-coumaric acid in the active site pocket of Y398S than in the wild-type enzyme [30]. In another protein engineering study of *Ab*HpaB [53], researchers replaced the R263 residue with Glu, Asp, or Ala to expand the substrate spectrum of the *Ab*HpaB enzyme. They found that the R263D mutant was a potent mutant capable of hydroxylating tyramine to form dopamine, with yields of 57%, whereas the wild type could not perform this conversion. To investigate whether the substrate utilization of R263D could be extended to other 4-hydroxyphenethylamine derivatives, researchers constructed double mutants. By creating a series of double mutants at the 263rd and 398th sites, they found that the mutant (R263D/Y398D) can catalyze the production of norepinephrine from octopamine, whereas the wild-type *Ab*HpaB was inactive against tyramine and octopamine [53]. Furthermore, engineering *Ab*HpaB to catalyze aniline was achieved in the S146A mutant, which can catalyze not only 4HPA but also 4-aminophenylacetic acid (4-APA, aniline derivatives). The S146A mutant could convert 4-APA to 3-hydroxy-4-aminophenylacetic acid at pH 6.0, with a yield of 100% compared with the 41% yield of the wild type. Notably, 4-APA binds to the wild-type enzyme only at pH 6.0, whereas it could bind to S146A at pH 6.0–9.0, with S146A hydroxylating 4-APA more efficiently at the lower pH. Moreover, density functional theory calculations of the binding energy indicated that 4-APA protonation favors ligand binding at a low pH. The primary change in S146A activity is the broadening of the substrate spectrum and pH range [54].

## 5. Applications of HpaB in the Synthesis of Catechol-Containing Compounds

The unique diversity of HpaB on substrates provides nearly limitless application potential to produce natural products, bioactive molecules, and pharmaceuticals that contain catechol motifs. Since its discovery, particularly in recent decades, HpaB from various bacteria has been successfully used for the synthesis of a series of flavones, stilbenes, monolignols, coumarins, and catecholamines, using either a direct phenol precursor via one-step biotransformation or using a simple carbon source via synthetic molecular pathways [46,47].

### 5.1. Synthesis of Catechol-Containing Compounds Using HpaB-Involved Synthetic Molecular Pathways

HpaB was widely used as a component in synthetic metabolic pathways to synthesize specific catechols from inexpensive and simple carbon sources, such as glucose and L-tyrosine. Given that L-tyrosine is the critical metabolite in the synthesis of phenols, the direct precursors of catechols, improving endogenous L-tyrosine synthesis and supplementing the media with exogenous L-tyrosine, were the most popular of those used to improve the production of catechol-containing compounds in related studies.

#### 5.1.1. Synthesis of Caffeic Acid and Its Derivatives

**Figure 7:**
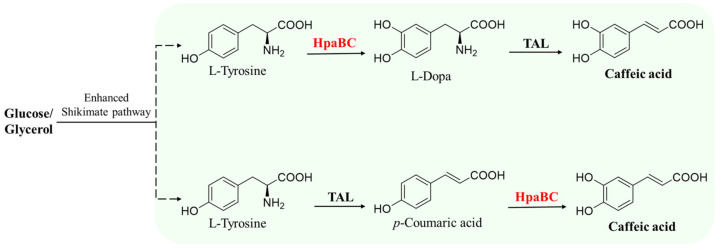
HpaB-involved synthetic molecular pathways for caffeic acid. TAL, tyrosine ammonia-lyase.

After identifying that native *Ec*HpaB exhibited moderate activity toward both *p*-coumaric acid and tyrosine, Lin et al. established the first artificial pathway in the *E. coli* strain for the de novo production of caffeic acid via the overexpression of *Ec*HpaB, *Ec*HpaC, and *Rhodobacter capsulatus* tyrosine ammonia-lyases (TALs) (Figure 7). The engineered *E. coli* strain could produce 12.1 mg·L^−1^ of caffeic acid from the carbon sources containing 10.0 g·L^−1^ glycerol and 2.5 g·L^−1^ of glucose in shake flasks after 48 h culture [55]. They further increased the caffeic acid titer in shake flasks (50.2 mg·L^−1^) by alleviating feedback inhibition and redirecting the carbon flux into tyrosine biosynthesis [55]. Moroever, they engineered a phenylalanine over-producer into a tyrosine over-producer and introduced their developed caffeic acid artificial pathway. After adjusting the expression strategy and optimizing the inoculant timing, the de novo production of caffeic acid from the engineered *E. coli* strain could attain 766.7 mg·L^−1^ in shake flasks after 72 h cultivation [16]. Liu et al. recruited HpaB and HpaC from several bacteria and constructed an artificial caffeic acid pathway similar to that in the Lin et al. study [55] (that is, overexpression of HpaB, HpaC, and TALs) in *Saccharomyces cerevisiae*. The highest production of caffeic acid from the engineered yeast was obtained with the enzyme combination of HpaB from *P. aeruginosa* and HpaC from *Salmonella enterica*, which yielded 289.4 mg·L^−1^ caffeic acid in shake flask cultivation. They also found that appropriate cooperation between HpaB and HpaC from different bacteria was extremely important in driving product synthesis from endogenous metabolic resources [56].

**Figure 8:**
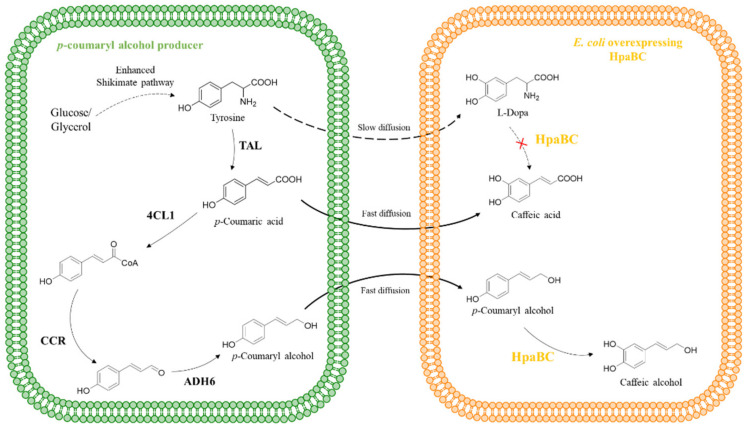
HpaB-involved synthetic molecular pathways for caffeic alcohol. TAL, tyrosine ammonia lyase; 4CL1, *p*-coumarate-CoA ligase; CCR, cinnamoyl-CoA reductase; ADH6, alcohol dehydrogenase.

Based on the successful construction of a biosynthetic pathway of *p*-coumaryl alcohol, Chen et al. [57] further extended the pathway to produce caffeic alcohol (Figure 8). The promiscuity of HpaB results in the formation of an unstable intermediate, L-dopa, from tyrosine, causing the loss of carbon sources during fermentation. To solve this problem, they adopted microbial cocultures of *p*-coumaryl alcohol producers and *E. coli* overexpressing HpaB and HpaC to minimize the accessibility of HpaB to tyrosine. They chose this strategy because the *E. coli* cell membrane has a lower diffusion resistance toward *p*-coumaryl alcohol compared to that of tyrosine. With the optimal inoculation ratio, 401.0 mg·L^−1^ of caffeic alcohol was produced. It is nearly 12 times higher than monoculture stain which was the *p*-coumaryl alcohol producer overexpressing HpaB and HpaC.

#### 5.1.2. Synthesis of Hydroxytyrosol Acid and Its Derivatives

**Figure 9:**
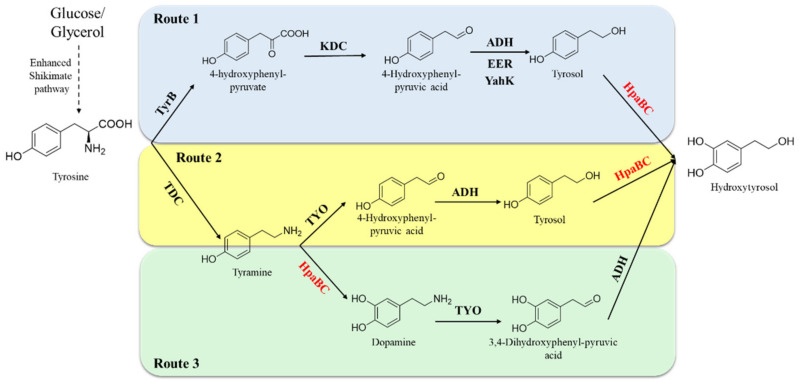
HpaB-involved synthetic molecular pathways for hydroxytyrosol. Route 1 (blue), Route 2 (yellow), and Route 3 (green). TyrB, aromatic-amino-acid aminotransferase; KDC, ketoacid decarboxylase; ADH, alcohol dehydrogenase; EER, endogenous *E. coli* reductase; YahK, aldehyde reductase; TDC; tyrosine decarboxylase; TYO, tyramine oxidase.

The development of highly efficient hydroxytyrosol-producing strains has always been pursued. HpaB exhibits considerable *ortho*-hydroxylation ability toward tyrosol (the direct phenol precursor of hydroxytyrosol); therefore, it is an excellent synthetic element to construct artificial pathways for hydroxytyrosol synthesis. Li et al. reported a synthesis pathway for de novo production of tyrosol from glucose and glycerol by overexpressing key enzymes (ketoacid decarboxylase and alcohol dehydrogenase) in the shikimate pathway and deleting the phenylacetaldehyde dehydrogenase gene (*feaB*) to prevent competition for the intermediate product, 4-hydroxyphenylacetaldehyde (4-HPAA). Next, native HpaB and HpaC were overexpressed to convert tyrosol into hydroxytyrosol [58] (Figure 9, Route 1). The combination of adding 1-dodecanol (to reduce hydroxytyrosol toxicity to the cells) and ascorbic acid (to decrease oxidation of hydroxytyrosol) and removing NH_4_Cl (to enhance transamination of tyrosine to 4-HPAA), enabled the final titer of hydroxytyrosol produced by the engineered *E. coli* to attain 1.2 g·L^−1^ from L-tyrosine in the feeding experiment and 647.0 mg·L^−1^ from simple carbon sources, respectively [58]. Choo et al. have developed another artificial hydroxytyrosol biosynthesis pathway based on HpaB [59] (Figure 9, Route 2). The pathways use a tyrosine decarboxylase from *Papaver somniferum* to convert L-tyrosine to tyramine, which is then oxidized to 4-HPAA by tyramine oxidase from *Micrococcus luteus.* 4-HPAA is spontaneously converted to tyrosol in *E. coli*, which is then hydroxylated to hydroxytyrosol by *E. coli* HpaB (Figure 9, Route 2). By increasing tyrosine synthesis via overexpressing 2-dehydro-3-deoxyphosphoheptonate aldolase and chorismite mutase in the shikimate pathway and deleting the *feaB* gene, the engineered *E. coli* with this hydroxytyrosol biosynthesis artificial pathway (Figure 9, Route 2) could result in a total of 268.3 mg·L^−1^ of hydroxytyrosol after 30 h fermentation using glucose, with a productivity of 9 mg·L^−1^·h^−1^ [59]. Moreover, Chen et al. observed that certain enzymes in the hydroxytyrosol-producing artificial pathway developed above (Route 2) had multiple substrates, which indicates that tyrosine could be potentially routed to hydroxytyrosol along two pathways (Figure 9, Routes 2 and 3) using the same enzymes [33]. They found that the synthetic capacity of hydroxytyrosol is significantly improved when the two pathways work simultaneously compared to each individual pathway. However, the two pathways have bottlenecks at different steps: *ortho*-hydroxylation of tyrosol to hydroxytyrosol (Figure 9, Route 2) and *ortho*-hydroxylation of tyramine to dopamine (Figure 9, Route 3). To overcome these challenges and enhance the efficiency of both pathways, the *Ec*HpaB mutant^S210T/A211M/Q212G^ which enhanced activity toward both tyrosol and tyramine was developed and involved in the two pathways. Engineered *E. coli* with mutant^S210T/A211M/Q212G^ involved in Routes 2 and 3 provided 1.9 g·L^−1^ hydroxytyrosol from tyrosine intracellularly with a yield of 82% in a 36 h experiment and exhibited more efficient productivity (52.5 mg·L^−1^·h^−1^) compared with the previous studies that produced hydroxytyrosol from simple sources [33]. The results indicate the promising and sustainable production of hydroxytyrosol and show a wide potential.

**Figure 10:**
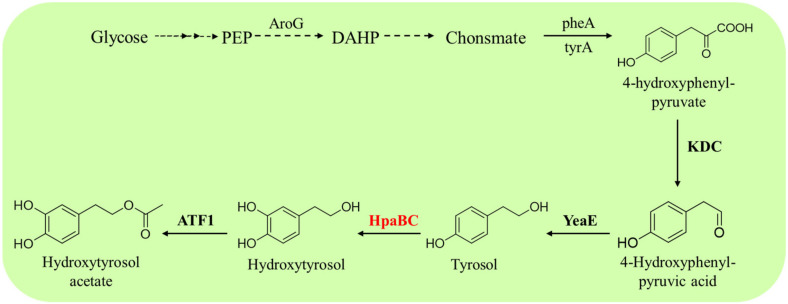
HpaB-involved synthetic molecular pathways for hydroxytyrosol acetate. AroG, 3-deoxy-D-arabinoheptulosonate-7-phosphate synthase; TyrA, chorismate mutase/prephenate dehydrogenase; KDC, 2-keto acid decarboxylase; YeaE, aldehyde reductases; ATF1, alcohol acetyltransferase.

Guo et al. produced hydroxytyrosol acetate from glucose by overexpressing HpaBC from *E. coli* and alcohol acetyltransferase (ATF1) from *S. cerevisiae* in a tyrosol-producing *E. coli* strain [60] (Figure 10). HpaBC catalyzes the hydroxylation of the generated tyrosol to hydroxytyrosol and ATF1, which catalyzes the acetylation of hydroxytyrosol to hydroxytyrosol acetate. The engineered *E. coli* could produce 225.0 mg·L^−1^ hydroxytyrosol acetate from glucose within 28 h [60]. This study represents a promising alternative to producing hydroxytyrosol acetate from renewable resources.

#### 5.1.3. Synthesis of Other Catechol-Containing Compounds

**Figure 11:**
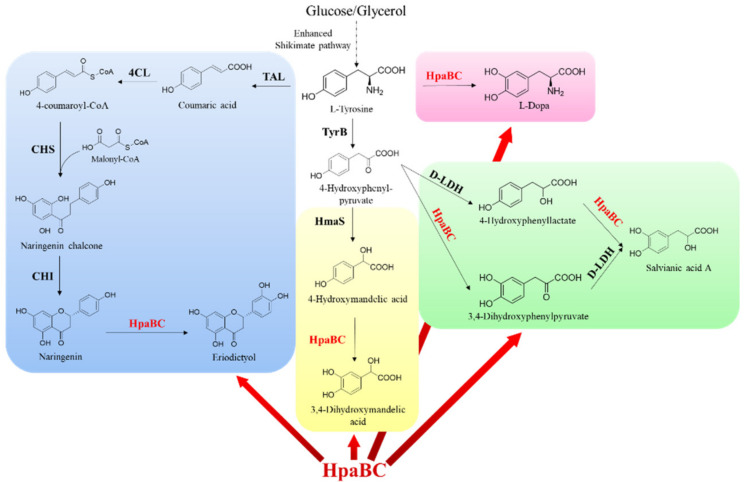
HpaB-involved synthetic molecular pathways for other catechol-containing compounds. The synthesis route of eriodictyol (blue zone): TAL, tyrosine ammonia lyase; 4CL, 4-coumaroyl-CoA ligase; CHS, chalcone synthase; and CHI, chalcone isomerase. The synthesis route of L-dopa (rose zone). The synthesis route of 3,4-dihydroxymandelic acid (yellow zone): HmaS, hydroxymandelate synthase. The synthesis route of salvianic acid A (green zone): D-LDH, D-lactate dehydrogenase.

HpaB and its mutants have also been used as synthetic components to construct artificial pathways for the synthesis of other catechol-containing compounds. Fordjour et al. constructed an engineered *E. coli* strain realized to produce 3,4-dihydroxyphenyl-L-alanine (L-dopa) from D-glucose via overexpressing the HpaB^G295R^ mutant, which showed an activity of approximately 3.0 times higher than the wild type, in a developed stain with high L-tyrosine yield. The engineered *E. coli* strain could produce 25.5 g·L^−1^ of L-dopa from glucose in a 5 L bioreactor in 48 h fermentation [61] (Figure 11, Rose zone). Their study achieved a higher titer than previous studies on de novo production of L-dopa with glucose or glycerol as the carbon source. In addition, by expressing *E. coli* HpaBC, Kanthasamy et al. generated a safe and tolerable probiotic bacterium, which synthesized L-dopa from tyrosine produced by the body as a live biotherapeutic agent to treat Parkinson’s disease [62]. This new approach also eliminated the side effects that develop when L-dopa is administered orally.

Li et al. achieved glucose-based biosynthesis of 3,4-dihydroxymandelic acid by overexpressing the hydroxymandelate synthase gene from *Streptomyces coelicolor* and the native HpaB and HpaC genes in *E. coli* MG1655/ΔA, which was a mutant developed in their laboratory to enhance the synthesis of 4-hydroxyphenylpyruvate (the precursor of 3,4-dihydroxymandelic acid) (Figure 11, Yellow zone). Under the optimized induction conditions, the yield of 3,4-dihydroxymandelic acid attained was 240 mg·L^−1^ in a shake flask after 36 h fermentation [63].

Yao et al. developed a novel artificial biosynthetic pathway for synthesizing salvianic acid A from glucose in *E. coli* via using native HpaB and HpaC and *Lactobacillus pentosus* D-lactate dehydrogenase mutant D-LDH^Y52A^ [64] (Figure 11, Green zone). Using a modular engineering approach and deleting genes involved in the regulatory and competing pathways to optimize the biosynthetic pathway, the developed metabolically engineered *E. coli* strain produced 7.1 g·L^−1^ salvianic acid A in 70 h fermentation, with a yield of 0.5 mol·mol^−1^ glucose [64], which exhibits a good prospect for industrialization.

In a recent study, Xia Wu et al. realized the biosynthesis of the *ortho*-hydroxylated flavone eriodictyol from L-tyrosine in *Corynebacterium glutamicum* for the first time via functional expression of *E. coli* HpaB and HpaC in naringenin producing *C. glutamicum* (Figure 11, Blue zone). Through optimization of the biotransformation process parameters, the engineered strain could produce 14.1 mg·L^−1^ eriodictyol [65]. The study provided new possibilities for the biosynthesis of plant flavones in this GRAS microorganism.

Using HpaB in synthetic molecular pathways enables the production of catechol-containing compounds from simple and inexpensive substrates. However, this strategy has proven difficult for achieving some catechol-containing compounds in high yields and titers, due to the demands of glucose and tyrosine for various other cellular activities.

### 5.2. One-Step Biotransformation to Catechol-Containing Compounds Using Direct Phenol Precursors

Direct phenol precursors are not central metabolites and allow the production of catechol-containing compounds in less enzymatic steps, offering the potential for higher yields. Since certain phenol precursors, such as *p*-coumaric acid and tyrosol, are inexpensive and easily obtained, the production of catechol-containing compounds from these precursors is attractive for industrialization (Table S2). When using phenols as synthetic precursors, both whole-cell biocatalysts (active cells in fermentation or resting cell conversion) and free enzyme systems have been used [15,28,30,31,36,66]. Among the related practices, a whole-cell biocatalyst is the most widely used, not only because it is simple, efficient, and economical but also because it can supply and regenerate the required cofactors, such as FAD/FADH_2_ and NAD(P)/NAD(P)H [15].

*ortho*-Hydroxylating phenol precursors in the fermentation process is a simple and convenient whole cell catalytic strategy to produce catechol-containing compounds. Through optimization of fermentation conditions, 62.7 mg·L^−1^ eriodictyol was produced from 300 mg·L^−1^ naringenin by using *E. coli*-overexpressed native HpaB and HpaC (BL21star^TM^(DE3)-pETM6/HpaBC) in Jones et al.’s study [66]. The authors also reported that the recombinant *E. coli* whole cells could *ortho*-hydroxylate afzelechin, affording catechin titers of 34.7 mg·L^−1^ [66]. Based on the optimization of culture conditions, including substrate concentration, induction temperature, substrate delay time, and medium types, Wang et al. successfully achieved the production of caffeic acid from *p*-coumaric acid, eriodictyol from naringenin, catechin from afzelechin, quercetin from kaempferol, and dihydroquercetin from dihydrokaempferol, with conversion rates of 33%, 58%, 35%, 24%, and 24%, respectively, by using the recombinant *E. coli* containing expression plasmids of pRSFDuet-*Ec*HpaBC and pETDuet-*Ec*HpaBC [32].

Compared with fermentation, resting cell conversion is another popular whole-cell catalytic strategy that reduces the difficulty in product purification. Lin et al. used cultured *E. coli* that overexpressed native HpaB and HpaC cells to *ortho*-hydroxylate of umbelliferone and resveratrol in M9Y medium containing 1.5 mM ascorbic acid; the production of esculetin and piceatannol could reach 2.7 and 1.2 g·L^−1^, respectively, with yields close to 100% [24]. Meanwhile, with the addition of 10% glycerol and 1% Tween-80, the resting cells of *E. coli*, which overexpressed *Pseudomonas aeruginosa* HpaB and HpaC, could produce 5.2 g·L^−1^ piceatannol by *ortho*-hydroxylation of resveratrol, with a conversion rate of 77%, which was the highest piceatannol yield via biotransformation reported [67]. Coulombel et al. employed recombinant *E. coli* Bl21(DE3)pETDuet-*Ec*HpaBC resting cells to *ortho*-hydroxylate of 4-halophenols. By keeping the ratio of the biocatalyst (*E. coli* CDW) to substrate concentration (mM) maintained at 2:1, 10.8 mM 4-fluorophenol was completely converted to the product within 7 h of conversion in a stirred-tank bioreactor. However, the conversion capacity of the whole-cell catalyst toward 4-fluoro-, 4-chloro-, 4-bromo-, and 4-iodophenol gradually decreases [31]. In a recent study, *E. coli* whole cells that overexpressed native HpaB and HpaC (cell density OD_600_ = 22) could produce 18.7 g·L^−1^ of caffeic acid from *p*-coumaric acid (conversion rate of 79%) in 6 h conversion at the optimized catalytic conditions with the addition of glucose at 1.7 g·L^−1^·h^−1^ [28]. To our knowledge, their titer of caffeic acid is the highest reported to date and the efficient process shows good application prospects for the large-scale production of caffeic acid.

Besides, there were some reports that to prepare catechols by using purified HpaB [30,53], for example using purified *Ab*HpaB^Y398S^, *Ab*HpaC, and glucose-6-phosphate dehydrogenase, 8.2 mg·L^−1^ *p*-coumaric acid could be completely converted to 3,4,5-trihydroxycinnamic acid within 180 min [30]. Chenprakhon et al. produced 0.175 g·L^−1^ dopamine from 2 mM tyramine by using purified *Ab*HpaB^R263D^ (50 μM), with a yield of 57% [53]. Both cases mentioned above used FMN^_^ as a cofactor for HpaB.

When using resting cells or isolated enzymes to convert phenol precursors, adopting appropriate methods to ensure an NAD(P)H supply for the HpaB and HpaC catalytic system is important. Thus far, the most widely used method is the addition of glucose or glycerol to the reaction buffer, which recycles NAD(P)H by metabolizing glucose or glycerin in bacterial cells [15,28,31]. In addition, NAD(P)H regeneration systems, such as formate dehydrogenase/formate and glucose-6-phosphate dehydrogenase/glucose-6-phosphate, were also used to supply NAD(P)H to the HpaB and HpaC catalytic system, which were proven to be successful [30,36].

## 6. Conclusions and Perspectives

HpaB is a vital component in the natural enzyme toolbox and has significant potential for application in catechol production. Recently, considerable efforts have been made to identify and engineer promising HpaB variants toward different substrates and develop effective synthesis systems to produce catechols and their derivatives; encouraging results have been obtained. However, few studies regarding the thermodynamic and operational stabilities of HpaB have been reported. The substrate selection mechanism of HpaB from different sources remains elusive. In addition, some of the discovered mutant activity toward certain substrates cannot satisfy industrialized applications. Since HpaB shows promiscuous activity toward substrates with closely similar structures, improving the HpaB specificity to enforce specific catechol compound synthesis in cell factories is challenging [29]. Moreover, the combinations of HpaB and HpaC from different species presented varied capabilities in producing the target product [56]. Therefore, it is important to analyze why special enzyme combinations work much better. Considering the importance of HpaB in catechols biosynthesis, further research on the functional mining of new HpaB sources, expanding its substrate scope; improving its properties, such as catalytic activity, product specificity, stability, and cofactor binding affinity; and improving the on-demand de novo design of HpaB to further enhance its application potential will be the frontiers of HpaB engineering. A systematic and in-depth study of the structure–function relationships in HpaB will provide valuable information for the engineering and use of HpaB in future biotechnological applications. In addition, new and effective artificial pathways, as well as robust HpaB whole-cell catalysts for catechols synthesis, require further research, which could create new and exciting opportunities for the practical catalysis of HpaB.

## Data Availability

The data presented in this study are available on request from the corresponding author.

## Supplementary Materials

The following supporting information can be downloaded at: https://www.mdpi.com/article/10.3390/molecules28186699/s1, Table S1: Examples and results of the molecular modifications of HpaB; Table S2: Examples of application of HpaB and its mutant to synthesis catechols using direct phenol precursors by one-step biotransformation.

## Sample Availability

Samples of the compounds are not available from the authors.
