# Supplementary material for: Advances in 4-Hydroxyphenylacetate-3-hydroxylase Monooxygenase
Source: Molecules. 2023 Sep 19;28(18):6699. doi: 10.3390/molecules28186699 (PMC10537072; doi:10.3390/molecules28186699)
Supplement: Supplementary file 1 [file molecules-28-06699-s001.zip › molecules-2606699-supplementary.pdf]

Table S1. Examples and results of the molecular modifications of HpaB.

Origins	Substrates	Products	Strategy Used	Mutation sites	Results	Reference
E. coli	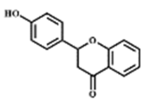 Naringenin	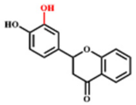 Eriodictyol	Structure-guided and enzyme mutagenesis	Loop (residues 207 to 217)	1.56-fold catalytic activity toward naringenin than the wild type.	[29]
A. baumannii	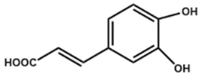 p-coumaric acid	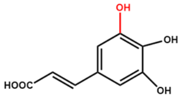 3,4,5-THCA	Molecular dynamics simulations, and site-directed mutagenesis	Y398S	Increased catalytic activity toward p-coumaric acid by 51% from 8%	[30]
E. coli	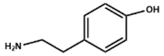 Tyramine	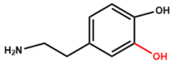 Dopamine	Molecular Docking, structure alignment, and enzyme mutagenesis	S210T/A211L/Q212E	271-fold catalytic activity toward tyramine than the wild type.	[33]
	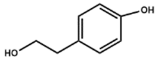 Tyrosol	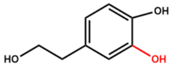 Hydroxytyrosol		S210T/A211L/Q212E	17-fold catalytic activity toward tyrosol than the wild type.	
	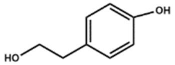 Tyrosol	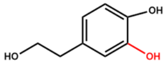 Hydroxytyrosol	Directed evolution	S210T/A211L/Q212E/Y282H	2.2-fold catalytic activity toward tyrosol than the wild type.	[37]

Table S1 (continued)

Origins	Substrates	Products	Strategy Used	Mutation sites	Results	Reference
E. coli	Tyrosine	L-DOPA	Modeling, and mutation approach	T15P/S210F/A211K/Q212F/D284E	15-fold catalytic activity toward tyrosine than the wild type.	[39]
R. opacus	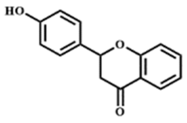 Naringenin	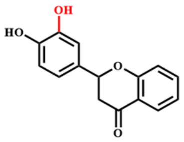 Eriodictyol	Molecular Docking, MD Simulation, and enzyme mutagenesis	Y215A	K_{cat}/K_m of naringenin catalyzed by the mutant Y215A increased by 25.3 times relative to the wild type.	[42]
E. coli	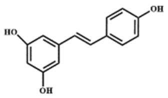 Resveratrol	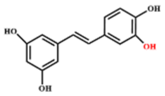 Piceatannol	Molecular Docking and saturation mutagenesis	I157L/A211D	4.7-fold catalytic activity toward resveratrol than the wild type.	[52]
A. baumannii	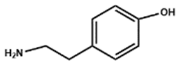 Tyramine	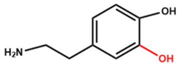 Dopamine	Site-directed mutagenesis	R263D	Expanding the substrate spectrum of *Ab*HpaB, *Ab*HpaB$^{R263D/Y398D}$ catalyzes tyramine	[53]
	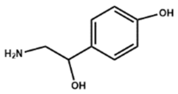 Octopamine	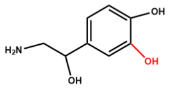 Norepinephrine		R263D/Y398D	Expanding the substrate spectrum of *Ab*HpaB, *Ab*HpaB$^{R263D/Y398D}$ catalyzes octopamine	

Table S1 (continued)

Origins	Substrates	Products	Strategy Used	Mutation sites	Results	Reference
A. baumannii	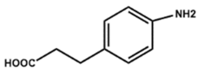 4-aminophenylacetic acid	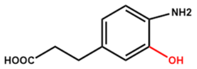 3-hydroxy-4-aminophenylacetic acid	Site-directed mutagenesis of amino acids near the substrate pocket	S146A	*Ab*HpaB^{S146A} increased catalytic 4-APA activity from 4% to 100%	[54]

Table S2 Examples of application of HpaB and its mutant to synthesis catechols using direct phenol precursors by one-step biotransformation

<table>
<tr><th>Method</th><th>Catalyts</th><th>Production condition</th><th>Reaction</th><th>Results</th><th>Reference</th></tr>
<tr><td rowspan="4">Fermntion</td><td rowspan="4">E. coli overexpressing native HpaBC (HpaBC expressed on double plamsids, BL21 (DE3)-pETDuet/hpaBC-pRSFDuet-EcHpaCB)</td><td rowspan="4">M9 medium, 80 mg·L^{-1} substrate added after induced with 1 mM IPTG at 28 °C for 6 h, then the fermentation lasted for 10 hours</td><td>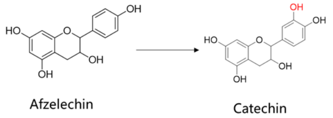
</td><td>29.81 mg·L^{-1} caffeic acid from 80 mg·L^{-1} afzelechin, 35.2% conversion rate</td><td rowspan="4">[32]</td></tr>
<tr><td>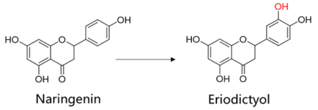
</td><td>46.84 mg·L^{-1} eriodictyo from 80 mg/L naringenin, 57.67 % conversion rate</td></tr>
<tr><td>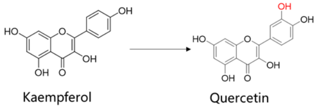
</td><td>20.14 mg·L^{-1} quercetin from 80 mg·L^{-1} kaempferol, 23.84 conversion rate</td></tr>
<tr><td>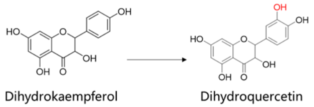
</td><td>20.05 mg·L^{-1} dihydroquercetin from 80 mg·L^{-1} dihydrokaempferol, 23.7% conversion rate</td></tr>
<tr><td rowspan="3">Fermntion</td><td rowspan="3">E. coli overexpressing native HpaBC (BL21star^{TM}(DE3)-pETM6/hpaBC)</td><td rowspan="3">Andrew's Magic Media (AMM) with 20 g·L^{-1} glycerol (For catechin, 20 g·L^{-1} glucose), substrate added after induced with 0.1 mM IPTG at 37 °C for 1 h (For caffeic acid and catechin, substrate after indcuded for 2 h), then the fermentation lasted for 48 hours</td><td>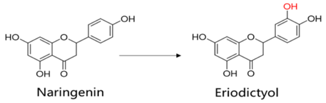
</td><td>62.7 mg·L^{-1} eriodictyol from 300 mg·L^{-1} naringenin, 19.74 % conversion rate</td><td rowspan="3">[66]</td></tr>
<tr><td>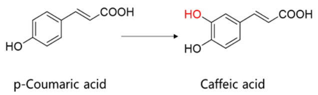
</td><td>3.46 g·L^{-1} caffeic acid from 4 g·L^{-1} p-coumaric acid, 78.82% conversion rate</td></tr>
<tr><td>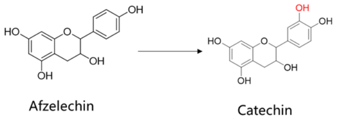
</td><td>34.7 mg·L^{-1} catechin from 300 mg·L^{-1} afzelechin, 10.93% conversion rate</td></tr>
</table>

Table S2 (continued)

Method	Catalyts	Production condition	Reaction	Results	Reference
Resting cells conversion	*E. coli* overexpressing native HpaBC (*E. coli* BW25113- pZE-HpaBC)	M9Y medium containing whole-cells (OD_{600}=9.6) and 1.5 mM ascorbic acid, convertion in shake flasks for 18 h with substate supplemention	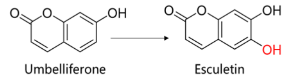<br>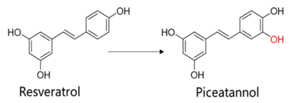	2.7 g·L^{-1} esculetin, close to 100% high yields.<br>1.2 g·L^{-1} piceatannol, close to 100% high yields	[24]
Resting cells conversion	*E. coli* overexpressing native HpaBC (BL21 (DE3)-pETDuet/hpaBC)	50 mM potassium phosphate buffer (pH 7.0) containing 10.4 g CDW/L whole cells and 4.8 mM substrate, convertion carried out in a 7.5-L stirred tank bioreactor at 30 °C for 3 h	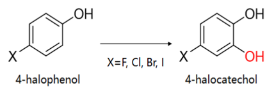	0.54g·L^{-1}(4.8 mM) 4-fluorophenol, with 100% conversion rate in 1.5 h. 0.62g·L^{-1} (4.8 mM) 4-Chlorophenol, 0.83g·L^{-1} (4.8 mM) 4-Bromophenol, 1.06g·L^{-1} (4.8 mM) 4-Iodophenol with 40%, 32% and 5% conversion rate, respectively	[31]
Resting cells conversion	*E. coli* overexpressing native HpaBC (BL21 (DE3)-pET28/hpaBC)	50 mM phosphate buffer (pH 7.0) with addition glucose and *p*-coumaric acid at a rate of 1.67 g·L^{-1}·h^{-1} and 5.4 g/(L·h), respectively, in a 5 L bioreactor at 37 °C for 6 h	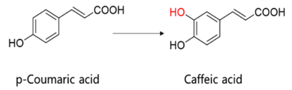	18.74 g·L^{-1} of caffeic acid, 78.81% conversion rate	[28]
Resting cells conversion	*E. coli* overexpressing HpaB^(S210T, A211L, Q212E, Y282H)C (*E. coli* MG1655-pEtac/HpaB^(S210T, A211L, Q212E, Y282H)C)	M9Y medium with 50 mM tyrosol and incubated at 200 rpm and 30 °C	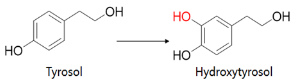	7.43 g·L^{-1} (48.2 mM) hydroxytyrosol converted from 6.91 g·L^{-1} (50 mM) tyrosol, 96.3% conversion rate	[37]

Table S2 (continued)

**Method**	**Catalyts**	**Production condition**	**Reaction**	**Results**	**Reference**
Resting cells conversion	*E. coli* overexpressing *Pseudomonas aeruginosa* HpaBC (BL21 (DE3)-pETDuet/hpaBC)	200 mM potassium phosphate buffer (pH 7.5) containing 32 g CDW/L whole cells, glycerol (10% v/v) and Tween 80 (1% v/v), convertion carried out at 30 ℃ in shake flasks for 12 h	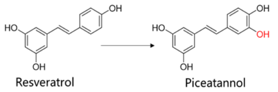	5.62 g·L^{-1} (23 mM) piceatannol from 6.85 g·L^{-1} (30 mM) resveratrol, 76.7% conversion rate	[67]
Enzyme	*Acinetobacter baumannii* HpaB mutant Y398S	The reaction carried out in a mixture of 1 mM glucose-6-phosphate, 100 nM HpaC from *Acinetobacter baumannii*, 10 μM NAD^+ , 1 μM FMN, 2 μM Y398S, 50 μM *p*-coumaric acid, 1.4 mM ascorbic acid, 0.5 unit·mL^{-1} glucose-6-phosphate dehydrogenase ml in 50 mM NaH_2PO_4 (pH 7.0) at room temperature for 180 min	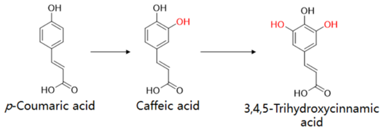	8.2 mg·L^{-1} (50 μM) *p*-coumaric acid could completely convert to 3,4,5-THCA within 180 min	[30]
